# Supplementary material for: A trans-oceanic flight of over 4,200 km by painted lady butterflies
Source: Nat Commun. 2024 Jun 25;15:5205. doi: 10.1038/s41467-024-49079-2 (PMC11199637; doi:10.1038/s41467-024-49079-2)
Supplement: Supplementary file 1 — Supplementary Information [file 41467_2024_49079_MOESM1_ESM.pdf]

Supplementary Information for

A trans-oceanic flight of over 4,200 km by painted lady butterflies

Tomasz Suchan†, Clément P. Bataille†, Megan S. Reich, Eric Toro-Delgado, Roger Vila, Naomi E. Pierce, Gerard Talavera†*

*Correspondence to: gerard.talavera@csic.es

This PDF file includes:

Tables S1 to S6
Figs. S1 to S5

Table S1. Mean interesection times to coastal West Africa from hourly computed backward wind trajectories, inferred by time frames between 6 a.m of 20th to 6 a.m 31st of October 2013. The percentage of trajectories reaching Africa is indicated. In bold we highlight the values for trajectories during 24h and 48h prior to the butterflies' observation in French Guiana.

	All altitudes		500 m AGL		1000 m AGL		2000 m AGL	
Time frame (24h)	Mean ± SE (h)	% traj.	Mean ± SE (h)	% traj.	Mean ± SE (h)	% traj.	Mean ± SE (h)	% traj.
21-22 Oct	-154.92 ± 2.82	33.3	NA	0	NA	0	-154.92 ± 2.82	100
22-23 Oct	-165.92 ± 0.98	33.3	NA	0	NA	0	-165.92 ± 0.98	100
23-24 Oct	-182.14 ± 1.30	29.3	NA	0	NA	0	-182.14 ± 1.30	88
24-25 Oct	NA	0	NA	0	NA	0	NA	0
25-26 Oct	NA	0	NA	0	NA	0	NA	0
26-27 Oct	-153.42 ± 1.69	82.7	-149.35 ± 1.13	92	-150.45 ± 0.63	88	-162.76 ± 5.38	68
**27-28 Oct**	**-164.52 ± 1.58**	**84**	**-170.52 ± 1.74**	**100**	**-165.32,3.23**	**76**	**-155.842 ± 2.42**	**76**
28-29 Oct	-188.25 ± 1.60	5.3	-188.25 ± 1.60	16	NA	0	NA	0
29-30 Oct	-152.04 ± 2.06	60	NA	0	-164.13 ± 0.80	92	-139.41 ± 1.63	88
30-31 Oct	-165.98 ± 2.62	57.3	NA	NA	-175 ± 2.75	76	-158.83 ± 3.57	96
	**All altitudes**		**500 m AGL**		**1000 m AGL**		**2000 m AGL**	
Time frame (48h)	Mean ± SE (h)	% traj.	Mean ± SE (h)	% traj.	Mean ± SE (h)	% traj.	Mean ± SE (h)	% traj.
20-22 Oct	-154.49 ± 2.01	27.9	NA	0	NA	0	-154.49 ± 2.01	83.67
22-24 Oct	-173.54 ± 1.46	31.3	NA	0	NA	0	-173.54 ± 1.46	93.88

24-26 Oct	NA	NA	NA	0	NA	0	NA	0
**26-28 Oct**	**-159.18 ± 1.28**	**83.0**	**-160.45 ± 1.90**	**95.92**	**-157.4 ± 1.96**	**81.63**	**-159.51 ± 2.91**	**71.43**
28-30 Oct	-155 ± 2.37	33.3	-188.25 ± 1.60	8.16	-164.13 ± 0.80	46.94	-139.41 ± 1.63	44.9

Table S2. Mean speed from computed 200h backward wind trajectories by time frames between 6 a.m of 22nd to 6 a.m 31st of October 2013. In bold we highlight the values used to interpret the trans-oceanic flight, including trajectories 48h prior to the butterflies' observation in French Guiana.

	**All altitudes**	**500 m AGL**	**1000 m AGL**	**2000 m AGL**
Time frame (24h)	Mean ± SE (m/s)	Mean ± SE (m/s)	Mean ± SE (m/s)	Mean ± SE (m/s)
21-22 Oct	7.36 ± 0.11	6.96 ± 0.07	6.78 ± 0.06	7.36 ± 0.11
22-23 Oct	7.32 ± 0.13	6.48 ± 0.16	6.68 ± 0.09	7.32 ± 0.13
23-24 Oct	6.29 ± 0.09	6.01 ± 0.09	5.90 ± 0.10	6.29 ± 0.09
24-25 Oct	7.11 ± 0.17	5.65 ± 0.14	7.28 ± 0.25	7.11 ± 0.17
25-26 Oct	6.20 ± 0.16	5.76 ± 0.20	5.71 ± 0.18	6.20 ± 0.16
26-27 Oct	7.74 ± 0.11	8.39 ± 0.07	8.22 ± 0.06	7.74 ± 0.11
27-28 Oct	7.21 ± 0.08	7.69 ± 0.08	7.33 ± 0.08	7.21 ± 0.08
28-29 Oct	7.44 ± 0.16	8.03 ± 0.23	8.08 ± 0.16	7.44 ± 0.16
29-30 Oct	8.01 ± 0.12	8.50 ± 0.28	8.10 ± 0.15	8.05 ± 0.12
30-31 Oct	8.34 ± 0.15	9.82 ± 0.17	8.64 ± 0.19	8.34 ± 0.15
Time frame (48h)	Mean ± SE (h)	Mean ± SE (h)	Mean ± SE (h)	Mean ± SE (h)
20-22 Oct	7.33 ± 0.07	6.85 ± 0.04	6.99 ± 0.05	7.33 ± 0.07
22-24 Oct	6.80 ± 0.09	6.24 ± 0.10	6.29 ± 0.08	6.81 ± 0.09
24-26 Oct	6.65 ± 0.12	5.73 ± 0.12	6.48 ± 0.17	6.65 ± 0.12
**26-28 Oct**	**7.47 ± 0.07**	**8.03 ± 0.07**	**7.77 ± 0.07**	**7.47 ± 0.07**
28-30 Oct	7.76 ± 0.10	8.27 ± 0.18	8.11 ± 0.11	7.76 ± 0.10

Table S3. Classification of plant ITS2 reads obtained from three *V. cardui* samples from French Guiana using Sickel *et al.* (2015)[1] and the PLANiTS[2] reference databases using SINTAX classifier[3] and following three different treatments: clustering at 98% (OTUs 0.98) and 99% (OTUs 0.99) sequence similarity, and with denoising using UNOISE algorithm (ZOTUs)[4]. Comments on plant species to which at least 100 sequences per sample were assigned are provided below the table.

	Sickel et al. 2015									PLANiTS								
	RVcoll14M973			RVcoll14M974			RVcoll14M975			RVcoll14M973			RVcoll14M974			RVcoll14M975		
species	OTUs 0.98	OTUs 0.99	ZOTUs	OTUs 0.98	OTUs 0.99	ZOTUs	OTUs 0.98	OTUs 0.99	ZOTUs	OTUs 0.98	OTUs 0.99	ZOTUs	OTUs 0.98	OTUs 0.99	ZOTUs	OTUs 0.98	OTUs 0.99	ZOTUs
Chelonanthus grandiflorus																4	4	4
Cucumis melo							6	6	6							6	6	6
Cynodon dactylon	6	6																
Foeniculum vulgare								5	14									
Guiera senegalensis [1]	11	11	11				21522	21451	19254	11	11	11				47480	47398	47495
Ipomoea imperati [2]										714	714	709	14	14	14	16	16	16
Ipomoea pes-caprae	48	48	33	16	16	12	7	7	7		15	15		11	11			
Mitracarpus frigidus [3]		8			506	4590			20									
Mitracarpus rigidifolius													22	3	25			
Passiflora foetida [4]	974	962	974								27	234						
Pseudostachyum polymorphum	3	3																
Pteris ensiformis				4	4	4												
Pteris tremula	10	46	46	4	4	4	2	15	12									
Pteris vittata [4]	13	3	10	1016	1014	984	5		5									
Tapirira guianensis	5	5	5							5	5	5						
Ziziphus spina-christi [4]				347	347	347												

[1] The most common OTUs were assigned to *Guiera senegalensis*, an African, Sahelian endemic. It was detected in two samples (RVcoll14M973, RVcoll14M975), using both reference databases and all three bioinformatic treatments. As the genus is monotypic, the classification should be robust, unlikely to belong to a closely-related species. The species, nor any closely related species were not handled in a laboratory where metabarcoding was performed.

[2] *Ipomoea imperati* - widespread tropical species, occuring on beaches, only detected using PLANiTS database[2] and in high numbers only in one specimen.

[3] *Mitracarpus frigidus* - up to 4590 reads in one specimen (depending on the bioinformatic pipeline) but only detected using Sickel et al. (2015)[1] database. With the PLANiTS database[2] some sequences were assigned to another closely-related species, *Mitracarpus rigidifolius*, also neotropical, but with a very low number of reads (up to 25).

[4] *Passiflora foetida* and *Pteris vittata* - OTUs assigned to these species were present only in one sample each and in high numbers only when using Sickel et al. (2015)[1] database; these are two widely distributed tropical species.

[5] *Ziziphus spina-christi*, an African endemic, was detected in one sample (RVcoll14M974) but only using Sickel et al. (2015)[1] database after all three bioinformatic treatments. However, all these OTUs (98% similarity OTU no 26; 98% similarity OTUs no 27, 186, 187, and 228; ZOTUs no 31, 39, 55, 58, and 94) had lowest E-value and highest sequence identity with ITS2 sequences of *Z. spina-christi* when comparing them using megablast with NCBI nucleotide database, indicating a good match.

Table S4. Hydrogen isotope values (transformed) and strontium isotope ratios (± measurement error) measured in the wings of the *V. cardui* butterfly samples.

Sample	δ^2H	$^{87}Sr/^{86}Sr$
14M973	-71 ± 3	0.71013 ± 0.00001
14M974	-68 ± 3	0.71084 ± 0.00001
14M975	-69 ± 3	0.71121 ± 0.00002

Table S5. Variables used for the energetic and flight model

Variable	Value	Organism	Source
w-butterfly weight	150mg	*Vanessa cardui*	Authors' unpublished data
Resting metabolic rate (RMR)	$0.4 – 0.6 ml \cdot O_2 \cdot g^{-1} \cdot h^{-1}$	*Danaus plexippus*	Parlin et al. (2023)[5]
Ratio of flight metabolic rate (FMR) to RMR	25 – 31	*Danaus plexippus*	Zhan et al. (2014)[6], Woods et al. (2005)[7]
Calories per ml of O_2	4.7	General	Parlin et al. (2023)[5]
mg of fat per calorie	0.11	General	Parlin et al. (2023)[5]
% fat from body weight	23	*Danaus plexippus*	Parlin et al. (2023)[5]
airspeed	6m/s	*Vanessa cardui*	Stefanescu *et al* (2013)[8]
windspeed	6.20 – 7.47m/s	Guyane – West Africa trajectories	This study

Table S6. Results of the energetic and flight duration models. Two different values of metabolic rate were used to calculate the duration of the fat reserves, considering a maximum possible fat content of 23% of body size. The models are inferred for a fully active flight strategy, where the metabolic rate is the active metabolic rate (AMR), and a "minimum-effort" flight strategy, where the metabolic rate is a weighted average of resting (85%) (RMR) and active (15%) metabolic rates. Both models consider a maximum possible fat content of 23% of body mass and two different metabolic rates depending on air temperature. The time required to cross the Atlantic is determined by integrating windspeeds from trajectories (mininum, average and maximum for October 26-28) and the airspeed of butterflies by self-powered flight, for both energetic scenarios. The LDD event is deemed energetically feasible (green values) only under a minimum-effort strategy. This strategy allows for the coverage of a maximum distance of ~7,000 km under the most favourable conditions of winds and metabolic rate. Conversely, unfeasible scenarios are represented by red values.

**Energetic flight model**							
	Self-powered flight speed (m/s)	6					
	Crossing distance (km)	4,200					
	Maximum fat content (%)	23					
	Wind speed (m/s)	4.71		7.47		8.79	
"minimum effort" strategy (85% RMR, 15% AMR)	Time to complete the crossing (hours)	208		139		120	
	Air temperature (ºC)	20	30	20	30	20	30
	Resting metabolic rate ($ml \cdot O_2 \cdot g^{-1} \cdot h^{-1}$)	0.4	0.6	0.4	0.6	0.4	0.6
	Active/resting metabolic rate ratio	31x	25x	31x	25x	31x	25x
	Averaged metabolic rate ($ml \cdot O_2 \cdot g^{-1} \cdot h^{-1}$)	2.2	2.76	2.2	2.76	2.2	2.76
	Required fat content (mg)	35.5	44.5	**23.8**	**29.8**	**20.5**	**25.8**
	Required fat content (%)	23.6	29.6	**15.8**	**19.9**	**13.7**	**17.2**
	Time to consume the maximum fat content (hours)	202	161	**202**	**161**	**202**	**161**
	Distance covered with maximum fat content (km)	4,086.8	3,257.6	**6,092.8**	**4,856.5**	**7,053.3**	**5,622.2**
Fully active flight (100% AMR)	Time to complete the crossing (hours)	109		87		79	
	Air temperature (ºC)	20	30	20	30	20	30
	Resting metabolic rate ($ml \cdot O_2 \cdot g^{-1} \cdot h^{-1}$)	0.4	0.6	0.4	0.6	0.4	0.6
	Active/resting metabolic rate ratio	31x	25x	31x	25x	31x	25x
	Active metabolic rate ($ml \cdot O_2 \cdot g^{-1} \cdot h^{-1}$)	12.4	15	12.4	15	12.4	15
	Required fat content (mg)	104.7	126.7	83.3	100.8	75.9	91.8
	Required fat content (%)	69.8	84.4	55.5	67.2	50.6	61.2
	Time to consume the maximum fat content (hours)	36	30	36	30	36	30
	Distance covered with maximum fat content, wind-assisted (km)	1,383.4	1,106.3	1,739.7	1,390.8	1,910.1	1,527.1
	Distance covered with maximum fat content, no wind (km)	774.9	619.6	774.9	619.6	774.9	619.6

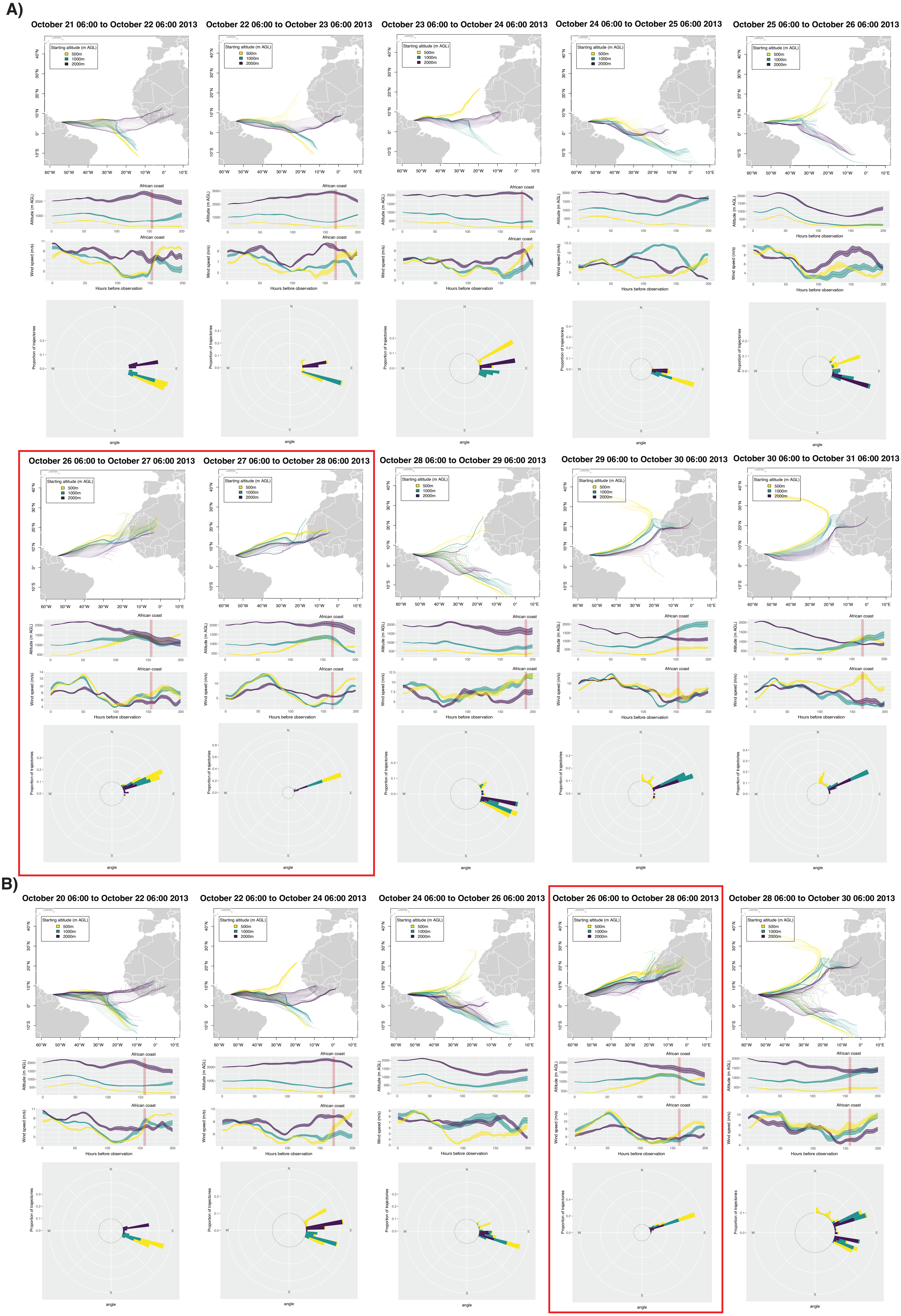
A)
October 21 06:00 to October 22 06:00 2013
October 22 06:00 to October 23 06:00 2013
October 23 06:00 to October 24 06:00 2013
October 24 06:00 to October 25 06:00 2013
October 25 06:00 to October 26 06:00 2013
October 26 06:00 to October 27 06:00 2013
October 27 06:00 to October 28 06:00 2013
October 28 06:00 to October 29 06:00 2013
October 29 06:00 to October 30 06:00 2013
October 30 06:00 to October 31 06:00 2013
B)
October 20 06:00 to October 22 06:00 2013
October 22 06:00 to October 24 06:00 2013
October 24 06:00 to October 26 06:00 2013
October 26 06:00 to October 28 06:00 2013
October 28 06:00 to October 30 06:00 2013
Starting altitude (m AGL)
500m
1000m
2000m
African coast
Altitude (m AGL)
Wind speed (m/s)
Hours before observation
Proportion of trajectories
angle

Fig. S1. Hourly backwards wind trajectories in **A)** 24 hours intervals from October 21st to October 31st 2013, or **B)** 48-hour intervals from October 20th to October 30th 2013 inferred using the Hybrid Single-Particle Lagrangian Integrated Trajectory (HYSPLIT) dispersion model based on the reanalysis database and computed for 200 hours at three altitudinal layers (500m, 1000m and 2000m above ground level). The top row displays the actual trajectories over the Atlantic. The middle rows shows the mean +- standard error of the altitude (in meters above ground level) and speed that the trajectories achieved at each hour of their duration, for each altitudinal layer. The bottom row shows circular histograms representing the proportion of trajectories (over the total in each 24-hour or 48-hour window) that, after the 200 hours, originate from a given compass direction (i.e. 0.5 indicates that half of the trajectories originate from that direction). Red boxes indicate trajectories 48h prior to the butterfly observation in French Guiana, on October 28th at 6 a.m.

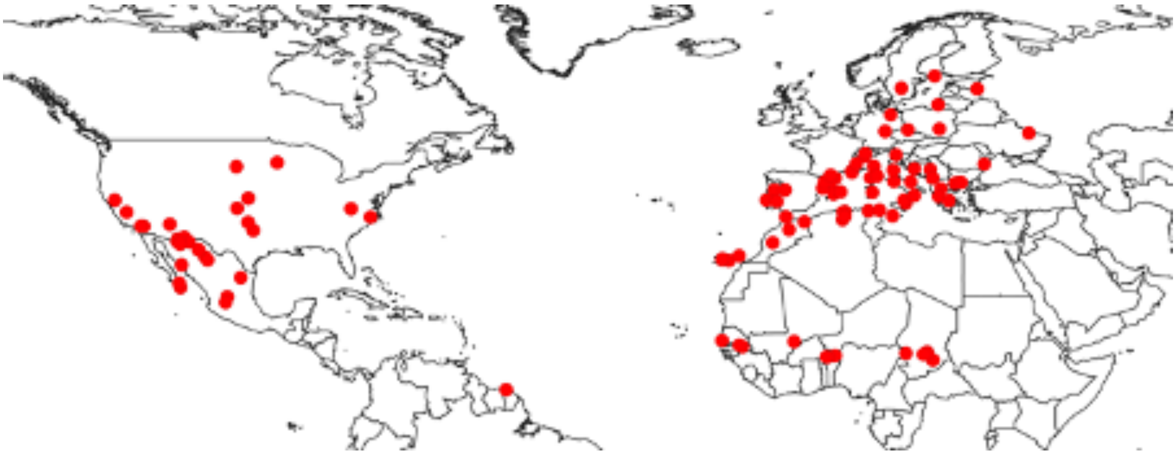

Fig. S2. Sampling distribution for ddRAD sequencing and population genomics analyses.

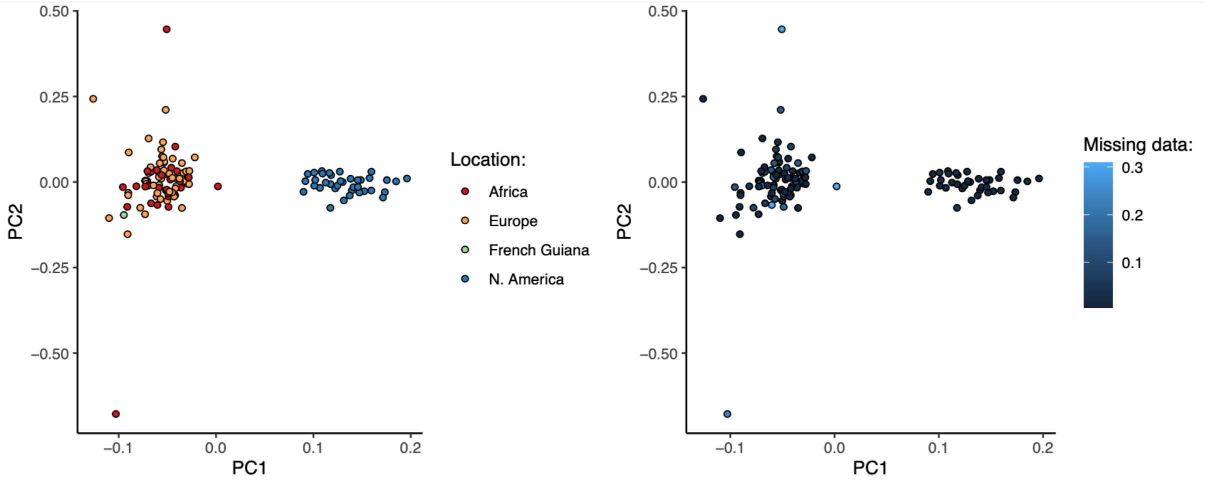

Fig. S3. Principal Component Analysis of RAD-seq derived SNPs, using only the unlinked variants with less than 10% of missing data (13 206 sites). Left plot shows the origin of the samples; the right plot shows the distribution of missing data per individual. An even distribution of missing data in the two genetic clusters indicates that the North America – Europe and Africa differentiation is not driven by the proportion in missing data, despite some outlier samples with less shared loci in Afican-European group.

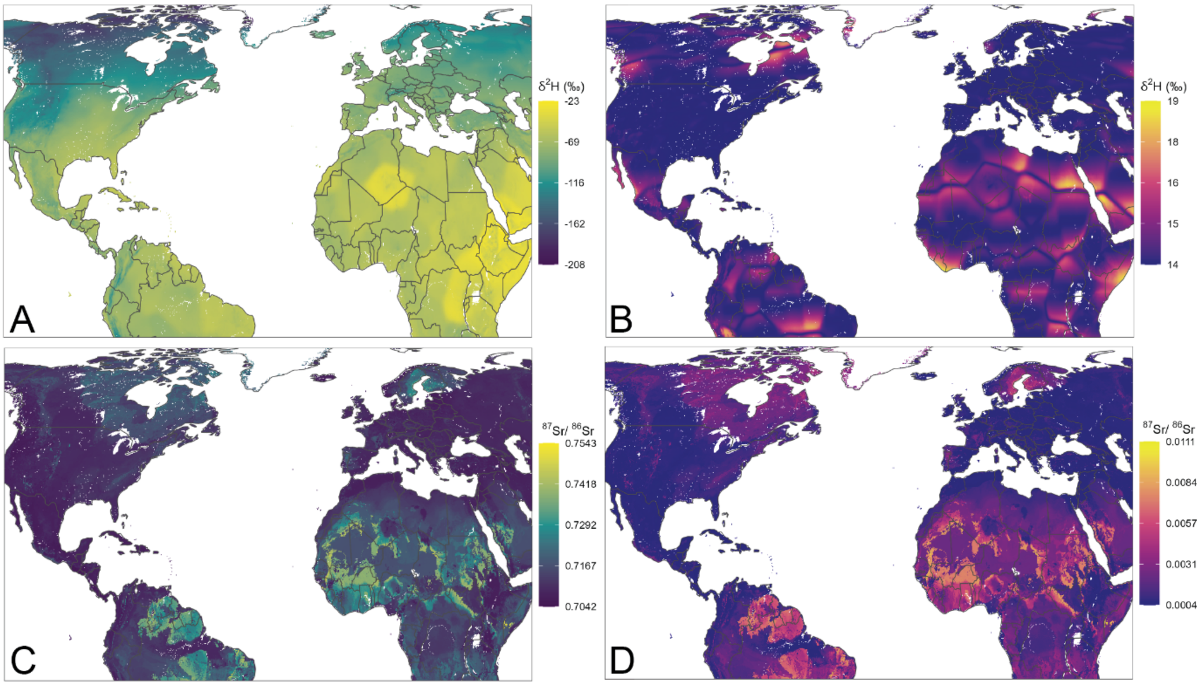

Fig. S4. Isoscapes used in the isotope-based geographic assignment. Butterfly wing hydrogen isoscape derived from mean annual precipitation isoscape (Bowen et al., 2005)[9] calibrated with known-origin data from Hobson et al. (2019)[10] and Ghouri et al. (2024)[11]: **A)** mean prediction (‰) and **B)** standard deviation. Global bioavailable strontium isoscape from Bataille et al., (2020)[12]: **C)** mean prediction and **D)** standard deviation.

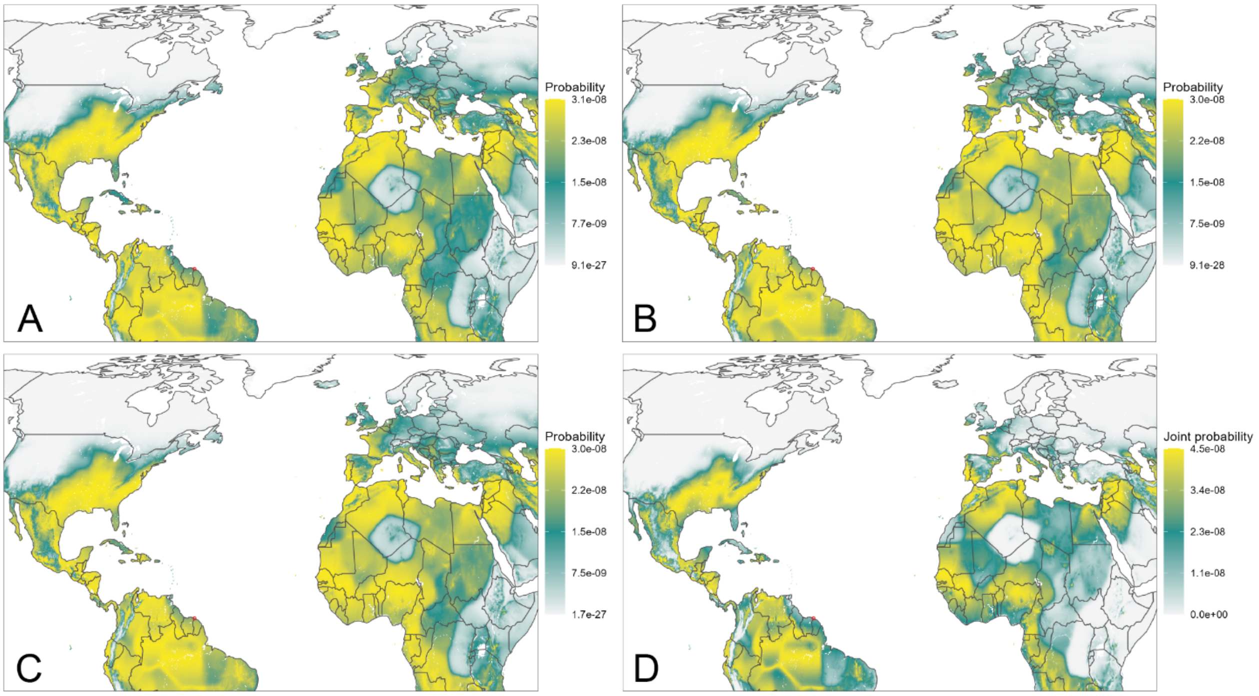

Fig. S5. Posterior probability surfaces of the hydrogen isotope-based geographic assignment for each individual: **A)** 14M973, **B)** 14M974, and **C)** 14M975. **D)** Joint probability surface (product of individual probabilities).

References

1. Sickel, W., et al. Increased efficiency in identifying mixed pollen samples by meta-barcoding with a dual-indexing approach. *BMC Ecology* **15**, 20 (2015).
2. Banchi, E., et al. PLANiTS: a curated sequence reference dataset for plant ITS DNA metabarcoding. *Database* **2020**, *baz155* (2020).
3. Edgar, R. C. SINTAX: a simple non-Bayesian taxonomy classifier for 16S and ITS sequences, *BioRxiv* (2016). https://doi.org/10.1101/074161
4. Edgar, R. C. UNOISE2: improved error-correction for Illumina 16S and ITS amplicon sequencing. *BioRxiv*, 081257 (2016). https://doi.org/10.1101/081257
5. Parlin, A. F., Kendzel, M. J., Taylor, O. R., Culley, T. M., Matter, S. F., & Guerra, P. A. The cost of movement: assessing energy expenditure in a long-distant ectothermic migrant under climate change. *Journal of Experimental Biology*, **226** (2023).

6. Zhan, S., Zhang, W., Niitepõld, K. *et al.* The genetics of monarch butterfly migration and warning colouration. *Nature* **514**, 317–321 (2014).
7. Woods, W. A., Jr. *Metabolic energy use by honeybees in flight and butterflies at rest.* (ProQuest Dissertations & Theses Global, 2005).
8. Stefanescu, C. et al. Multi-generational long-distance migration of insects: Studying the painted lady butterfly in the Western Palaearctic. *Ecography* **36**, 474–486 (2013).
9. Bowen, G. J., Wassenaar, L. I. & Hobson, K. A. Global Application of Stable Hydrogen and Oxygen Isotopes to Wildlife Forensics. *Oecologia* **143**, 337–348 (2005).
10. Hobson, K. A., Kardynal, K. J. & Koehler, G. Expanding the Isotopic Toolbox to Track Monarch Butterfly (*Danaus plexippus*) Origins and Migration: On the Utility of Stable Oxygen Isotope ($\delta^{18}O$) Measurements, *Frontiers in Ecology and Evolution* **7**, 224 (2019).
11. Ghouri, S., Reich, M. S., Lopez-Mañas, R., Talavera, G., Bowen, G. J., Vila, R., Talla, V. N. K., Collins, S. C., Martins, D. J., & Bataille, C. P. A hydrogen isoscape for tracing the migration of herbivorous lepidopterans across the Afro-Palearctic range. *Rapid Communications in Mass Spectrometry* **38**, e9675 (2024).
12. Bataille, C. P., Crowley, B. E., Wooller, M. J. & Bowen, G. J. Advances in global bioavailable strontium isoscapes, *Palaeogeography, Palaeoclimatology, Palaeoecology* **555**, 109849 (2020).
