# Supplementary material for: A trans-oceanic flight of over 4,200 km by painted lady butterflies
Source: Nat Commun. 2024 Jun 25;15:5205. doi: 10.1038/s41467-024-49079-2 (PMC11199637; doi:10.1038/s41467-024-49079-2)
Supplement: Supplementary file 3 — Reporting Summary [file 41467_2024_49079_MOESM3_ESM.pdf]

nature portfolio

Corresponding author(s): Gerard Talavera

Last updated by author(s): Apr 8, 2024

Reporting Summary

Nature Portfolio wishes to improve the reproducibility of the work that we publish. This form provides structure for consistency and transparency in reporting. For further information on Nature Portfolio policies, see our Editorial Policies and the Editorial Policy Checklist.

Statistics

For all statistical analyses, confirm that the following items are present in the figure legend, table legend, main text, or Methods section.

n/a	Confirmed	
☒	☐	The exact sample size (*n*) for each experimental group/condition, given as a discrete number and unit of measurement
☒	☐	A statement on whether measurements were taken from distinct samples or whether the same sample was measured repeatedly
☒	☐	The statistical test(s) used AND whether they are one- or two-sided<br>*Only common tests should be described solely by name; describe more complex techniques in the Methods section.*
☒	☐	A description of all covariates tested
☒	☐	A description of any assumptions or corrections, such as tests of normality and adjustment for multiple comparisons
☐	☒	A full description of the statistical parameters including central tendency (e.g. means) or other basic estimates (e.g. regression coefficient) AND variation (e.g. standard deviation) or associated estimates of uncertainty (e.g. confidence intervals)
☒	☐	For null hypothesis testing, the test statistic (e.g. *F*, *t*, *r*) with confidence intervals, effect sizes, degrees of freedom and *P* value noted<br>*Give P values as exact values whenever suitable.*
☒	☐	For Bayesian analysis, information on the choice of priors and Markov chain Monte Carlo settings
☒	☐	For hierarchical and complex designs, identification of the appropriate level for tests and full reporting of outcomes
☒	☐	Estimates of effect sizes (e.g. Cohen's *d*, Pearson's *r*), indicating how they were calculated

Our web collection on statistics for biologists contains articles on many of the points above.

Software and code

Policy information about availability of computer code

Data collection	no software was used to collect data
Data analysis	HYSPLIT<br>R (Rx64 4.3.2) packages: lubridate, splitr, opentraj66, sp, raster, geosphere, ggplot2, viridis, terra, assignR,<br>ipyrad v.0.9.81<br>fineRADstructure v.0.3.1<br>VCFtools v0.1.16<br>PLINK v1.90<br>CUTADAPT v3.2<br>VSEARCH v2.20.0<br>Custom code: https://github.com/GTlabIBB/Guyane (https://zenodo.org/doi/10.5281/zenodo.10901404)

For manuscripts utilizing custom algorithms or software that are central to the research but not yet described in published literature, software must be made available to editors and reviewers. We strongly encourage code deposition in a community repository (e.g. GitHub). See the Nature Portfolio guidelines for submitting code & software for further information.

Data

Policy information about availability of data

All manuscripts must include a data availability statement. This statement should provide the following information, where applicable:

- Accession codes, unique identifiers, or web links for publicly available datasets
- A description of any restrictions on data availability
- For clinical datasets or third party data, please ensure that the statement adheres to our policy

Demultiplexed and adapter-trimmed RAD-sequencing reads: European Nucleotide Archive project PRJEB57763 (https://www.ebi.ac.uk/ena/data/view/PRJEB57763). Raw metabarcoding sequences: European Nucleotide Archive project PRJEB57731 (https://www.ebi.ac.uk/ena/data/view/PRJEB57731). Scripts used to process the data: https://github.com/GTlabIBB/Guyane

Research involving human participants, their data, or biological material

Policy information about studies with human participants or human data. See also policy information about sex, gender (identity/presentation), and sexual orientation and race, ethnicity and racism.

Reporting on sex and gender	n/a
Reporting on race, ethnicity, or other socially relevant groupings	n/a
Population characteristics	n/a
Recruitment	n/a
Ethics oversight	n/a

Note that full information on the approval of the study protocol must also be provided in the manuscript.

Field-specific reporting

Please select the one below that is the best fit for your research. If you are not sure, read the appropriate sections before making your selection.

☐ Life sciences ☐ Behavioural & social sciences ☒ Ecological, evolutionary & environmental sciences

For a reference copy of the document with all sections, see nature.com/documents/nr-reporting-summary-flat.pdf

Ecological, evolutionary & environmental sciences study design

All studies must disclose on these points even when the disclosure is negative.

Study description	We studied 3 specimens of the butterfly Vanessa cardui collected outside their distribution range as potential long-range migrants. We combined pollen metabarcoding, phylogeography, stable isotopes and wind and energetic models to trace back their potential dispersal history and natal origin.
Research sample	3 individuals of Vanessa cardui, collected resting on the beach in French Guiana on October 28th, 2013 were collected for the molecular and isotopic component of this study. Molecular data for additional 128 individuals were obtained for phylogeographic assignment of French Guiana's samples.
Sampling strategy	The sample size was determined by the number of specimens that could be caught in the field.
Data collection	No data was collected in the field. Data were obtained from H and Sr isotope analyses from wing tissue, from DNA sequencing through ddRAD libraries, through metabarcoding of pollen grains, and from modelling wind trajectories.
Timing and spatial scale	Surveys for the butterflies were performed in October 2013 form sunrise, starting around 6 a.m. until about 10 a.m. On October 28th, 2013, V. cardui butterflies were found with open wings standing in the sand, just a few meters from the water line.
Data exclusions	No data were excluded from the analyses.
Reproducibility	The input files used for our analyses are provided as supplementary Data for the purposes of reproducibility.
Randomization	Not relevant to the study - the dataset consisted of three samples, limited by the number of specimens that could be caught in the field. No experimental procedures were involved.

Blinding	Our work was not experimental and thus blinding is not applicable. The software used for wind modelling, phylogeographic assigments, isotope geolocation and pollen-based plant identification do not provide information on previous hypotheses and it interprets the dataset strictly based on the input data files.

Did the study involve field work? ☒ Yes ☐ No

Field work, collection and transport

Field conditions	Surveys were done along beaches in French Guiana during the morning.
Location	French Guiana, 5.745902 N, 53.933677 W
Access & import/export	Collecting sites were not protected areas and access was not restricted. At the time of sampling no permits were required to sample outside National Parks in French Guiana (an overseas department of France). The specimens were collected before enforcement of the Nagoya protocol and collected outside of protected areas, thus not requiring collection and export permits.
Disturbance	Collecting the samples did not cause any disturbance to the environment.

Reporting for specific materials, systems and methods

We require information from authors about some types of materials, experimental systems and methods used in many studies. Here, indicate whether each material, system or method listed is relevant to your study. If you are not sure if a list item applies to your research, read the appropriate section before selecting a response.

Materials & experimental systems

n/a	Involved in the study	
☒	☐	Antibodies
☒	☐	Eukaryotic cell lines
☒	☐	Palaeontology and archaeology
☐	☒	Animals and other organisms
☒	☐	Clinical data
☒	☐	Dual use research of concern
☒	☐	Plants

Methods

n/a	Involved in the study	
☒	☐	ChIP-seq
☒	☐	Flow cytometry
☒	☐	MRI-based neuroimaging

Animals and other research organisms

Policy information about studies involving animals; ARRIVE guidelines recommended for reporting animal research, and Sex and Gender in Research

Laboratory animals	No laboratory animals were used in the study.
Wild animals	3 individuals of Vanessa cardui, collected and killed in the field.
Reporting on sex	n/a
Field-collected samples	3 individuals of Vanessa cardui, collected and immediately killed in the field.
Ethics oversight	No ethical approval was required for working with non-endangered invertebrate specimens.

Note that full information on the approval of the study protocol must also be provided in the manuscript.

Plants

Seed stocks	n/a
Novel plant genotypes	n/a
Authentication	n/a
