# Supplementary material for: A trans-oceanic flight of over 4,200 km by painted lady butterflies
Source: Nat Commun. 2024 Jun 25;15:5205. doi: 10.1038/s41467-024-49079-2 (PMC11199637; doi:10.1038/s41467-024-49079-2)
Supplement: Supplementary file 4 — Description of Additional Supplementary Files [file 41467_2024_49079_MOESM4_ESM.pdf]

Description of Additional Supplementary Files

File Name: Supplementary Data 1

Description: Sampling used for molecular analyses.

File Name: Supplementary Data 2

Description: The number of raw reads, number of reads passed filters (with the proper sample barcode and restriction enzyme cutsites present), number of reads mapped and unmapped on the reference genome, number of clusters and high-coverage clusters per sample, estimated heterozygosity and error rates, consensus clusters in each sample and the final number of loci in the assembly.
